# Response to: Comment on “Hepatitis C and the Sex Trade”

**DOI:** 10.1155/2016/5474217

**Published:** 2016-06-28

**Authors:** Stephen D. Shafran

**Affiliations:** Division of Infectious Diseases, Department of Medicine, University of Alberta, Edmonton, AB, Canada T6G 2G3

I thank Socías and colleagues for their letter [1] in response to my editorial [2].

Socías et al. continue to claim that female sex workers are at significant risk of acquiring HCV infection via sexual contact, citing a study by Feldman et al. [3]. Despite the declarative title, the data in the study of Feldman et al. do not support sexual transmission of HCV, nor do they support permucosal transmission. Feldman et al. found that 8 of 502 urban mainly (91%) Black women aged 17 to 49 years, 53% of whom were born outside the United States, tested positive for anti-HCV, for a seroprevalence of 1.6%, very similar to the seroprevalence of anti-HCV of 1.9% (381/20,042) in Americans aged ≥20 years in the much larger NHANES study [4]. Feldman et al. reported that 5 of the 8 anti-HCV positive women used drugs in the past year but provide no data regarding injection drug use more than one year prior to HCV testing in the study or information regarding parenteral medication use or vaccine administration in their countries of birth, all of which can transmit HCV infection.

In addition to the HCV Partners Study, cited in my editorial, in which the rate of heterosexual transmission of HCV was estimated at 1 in 190,000 sexual contacts [5], multiple other studies attest to the rarity of heterosexual transmission of HCV [6–10].

Socías et al. write that my original editorial overlooked “the significant limitations of risk-based testing.” I respectfully disagree. My editorial stated that the vast majority of HCV cases will be identified testing persons with a history of having ever used injection drugs and/or an elevated ALT, and that statement remains true. I never claimed that risk-based testing would identify all HCV cases, because it will not. I do not know why Socías et al. discuss the limitations of “symptomatic testing,” which I did not advocate, since few HCV-infected patients have symptoms that suggest the diagnosis of HCV infection. On that point, we agree completely.

Their statement that “ALT levels are typically within the normal range in up to 40% of people with chronic HCV” is also untrue. They support that statement by referencing a publication that actually states that the prevalence of normal ALT in chronic HCV infection is 20–30% [11], which in turn cites two studies that do not provide an ALT cutoff for the upper limit of normal [12, 13]. It is clear that the prevalence of normal ALT in chronic HCV infection is entirely dependent on the ALT cutoff chosen for the upper limit of normal. When updated upper limits of normal values for ALT of 30 U/L in men and 19 U/L in women are used [14], the proportion of patients with chronic HCV and normal ALT is quite low, and when followed over time, most of these patients have intermittently elevated ALT [15].

I make no apology for stating that adult patients need to take personal responsibility for their health, with the proviso that they are mentally competent to do so. I did not and do not use the word “blame” hurled at me by Socías et al. I completely deny the offensive accusation that my “editorial suggests that HCV treatment should be discouraged among women involved in sex work.” The editorial makes no such suggestion and I have personally treated female sex workers for HCV infection and will continue to do so.
